# ﻿Morphological and molecular phylogenetic analyses reveal three species of *Colletotrichum* in Shandong province, China

**DOI:** 10.3897/mycokeys.85.75944

**Published:** 2021-12-08

**Authors:** Taichang Mu, Zhaoxue Zhang, Rongyu Liu, Shubin Liu, Zhuang Li, Xiuguo Zhang, Jiwen Xia

**Affiliations:** 1 Shandong Provincial Key Laboratory for Biology of Vegetable Diseases and Insect Pests, College of Plant Protection, Shandong Agricultural University, Taian, 271018, China Shandong Agricultural University Tai'an China

**Keywords:** *Colletotrichum*, Glomerellaceae, multi-gene phylogeny, new species, taxonomy

## Abstract

*Colletotrichum* has numerous host range and distribution. Its species are important plant pathogens, endophytes and saprobes. *Colletotrichum* can cause regular or irregular depressions and necrotic lesions in the epidermal tissues of plants. During this research *Colletotrichum* specimens were collected from Mengyin County, Shandong Province, China. A multi-locus phylogenetic analysis of ITS, GAPDH, CHS-1, ACT, TUB2, CAL and GS sequence data combined with morphology, revealed a new species and two known species, viz. *C.mengyinense* sp. nov., *C.gloeosporioides* and *C.pandanicola*, belonging to the *C.gloeosporioides* species complex. The new species is described and illustrated in this paper and compared with taxa in the *C.gloeosporioides* species complex.

## ﻿Introduction

*Colletotrichum* species (Glomerellaceae, Glomerellales) is one of the ten economically most important fungal plant pathogens worldwide (Dean et al. 2012). It was first observed by Tode (1790), who divided it into *Vermicularia*. Corda (1831) established *Colletotrichum* based on the characteristic of the conidiomata with setae in *Vermicularia*. *Colletotrichum* is based on the type species *Colletotrichumlineola* which was associated with a member of the *Apiaceae* (Jayawardena et al. 2017). The sexual morph belongs to *Glomerella*. The asexual morph is characterized by acervuli born in the skin of the host, often producing brown sharp setae, colorless or brown conidiophores with separate, conidia colorless, pseudomonas, cylindrical or crescent-shaped (Damm et al. 2009).

Currently, more than 900 epithets of *Colletotrichum* are listed in Index Fungorum (http://www.indexfungorum.org/; accessed 22 November 2021). *Colletotrichum* has been studied for more than 200 years and the classification of *Colletotrichum* has undergone major changes (Jayawardena et al. 2016). In order to clarify its complex nature, the species are classified into 14 species complexes (Bhunjun et al. 2021). Specifically, *C.gloeosporioides* has been considered as a complex species for a long time.

The name *C.gloeosporioides* was first proposed by Penzig based on *Vermiculariagloeosporioides* which was collected from *Citrus* in Italy (Weir et al. 2012). Early in the study of *C.gloeosporioides* species complex, taxonomic concepts used were based on apparent features such as morphological characters, host species, size and shape of conidia and appressoria, presence or absence of setae, aspect, color and growth rate in culture, whether or not the teleomorph develops, etc (Weir et al. 2012). Nonetheless, Sutton commented that “no progress in the systematics and identification of isolates belonging to this complex is likely to be made based on morphology alone”. Fortunately, with the development of molecular systematics, gene method is applied to taxonomy of *Colletotrichum* complexes. Multi-gene phylogeny analysis is of great significance to the study of the classification of *C.gloeosporioides* species complex and related concepts of species (Cannon et al. 2012; Damm et al. 2012; Weir et al. 2012).

The aim of this study was to explore the diversity of *Colletotrichum* species from symptomatic leaves and diseased fruit of plants in Shandong Province, China. We present a new species and two known species, *C.mengyinense* sp. nov., *C.gloeosporioides* and *C.pandanicola* based on phylogenetic data and morphology.

## ﻿Materials and methods

### ﻿Isolation and morphological studies

The samples were collected from Mengyin County, Shandong Province, China. The strains of *Colletotrichum* were isolated from symptomatic leaves of *Rosachinensis* and diseased fruit of *Juglansregia* using single spore and tissue isolation methods (Chomnunti et al. 2014). The spore suspension was obtained and spread onto PDA plate and incubated for one day under the biochemical incubator. After germination, the spores were transferred to a new PDA plate to obtain pure culture. Additionally, the surface sterilized plant tissue isolation was used to obtain sterile isolates from the host plant. About 25 mm^2^ tissue fragments were taken from the margin of tissue lesions and surface sterilized by consecutively immersing in 75% ethanol solution for 60 s, 5% sodium hypochlorite solution for 30 s, and then rinsed in sterile distilled water for 60 s (Gao et al. 2013; Liu et al. 2015). The surface sterilized plant tissue was dried with sterilized paper and moved on the PDA plate (Cai et al. 2009). All the PDA plates were incubated at biochemical incubator at 25 °C for 3–4 days, then hyphae were picked out of the periphery of the colonies and inoculated on to new PDA plates.

Following 5–14 days of incubation, morphological characters were recorded (Cai et al. 2009). Photographs of the colonies were taken at 7 days and 14 days using a digital camera (Canon G7X). Micromorphological characters of colonies were observed using stereomicroscope (Olympus SZX10) and microscope (Olympus BX53), both fitted with high definition color digital cameras to photo document conidia and so on of fungal structures. All *Colletotrichum* strains were stored in 10% sterilized glycerin and sterile water at 4 °C for deep studies in the future. Every specimen was deposited in the Herbarium of the Department of Plant Pathology, Shandong Agricultural University (HSAUP). Living cultures were deposited in the Shandong Agricultural University Culture Collection (SAUCC). Taxonomic information of the new taxa was submitted to MycoBank (http://www.mycobank.org).

### ﻿DNA extraction and amplification

Genomic DNA was extracted from *Colletotrichum* fungal mycelia grown on PDA after 5–7 days, using a modified cetyltrimethylammonium bromide (CTAB) buffer, and then it was incubated at 65 °C for 30 min with occasional gentle inverting (Guo et al. 2000). Gene sequences were obtained from seven genes loci including the internal transcribed spacer regions with intervening 5.8S nrRNA gene (ITS), partial glyceraldehyde-3-phosphate dehydrogenase gene (GAPDH), partial chitin synthase 1 gene (CHS-1), partial actin gene (ACT), partial beta-tubulin gene (TUB2), partial calmodulin gene (CAL) and partial glutamine synthetase gene (GS) were amplified and sequenced using primers pairs (Table 1).

**Table 1.** Gene regions and respective primer pairs used in the study.

Locus	Gene	Primer	Direction	Sequence (5'-3')
The internal transcribed spacer regions with intervening 5.8S nrRNA gene	ITS	ITS5	Forward	GGA AGT AAA AGT CGT AAC AAG G
		ITS4	Reverse	TCC TCC GCT TAT TGA TAT GC
Partial glyceraldehyde-3-phosphate dehydrogenase gene	GAPDH	GDF1	Forward	GCC GTC AAC GAC CCC TTC ATT GA
		GDR1	Reverse	GGG TGG AGT CGT ACT TGA GCA TGT
Partial chitin synthase 1 gene	CHS-1	CHS-79F	Forward	TGG GGC AAG GAT GCT TGG AAG AAG
		CHS-354R	Reverse	TGG AAG AAC CAT CTG TGA GAG TTG
Partial actin gene	ACT	ACT-512F	Forward	ATG TGC AAG GCC GGT TTC GC
		ACT-783R	Reverse	TAC GAG TCC TTC TGG CCC AT
Partial beta-tubulin gene	TUB2	Bt-2a	Forward	GGT AAC CAA ATC GGT GCT GCT TTC
		Bt-2b	Reverse	ACC CTC AGT GTA GTG ACC CTT GGC
Partial calmodulin gene	CAL	CL1	Forward	GAR TWC AAG GAG GCC TTC TC
		CL2A	Reverse	TTT TTG CAT CAT GAG TTG GAC
		CL1C	Forward	GAA TTC AAG GAG GCC TTC TC
		CL2C	Reverse	CTT CTG CAT CAT GAG CTG GAC
Partial glutamine synthetase gene	GS	GSLF3	Forward	GAT ACG CCT CTT CCA GCG TT
		GSLR1	Reverse	AGR CGC ACA TTG TCA GTA TCG

PCR was performed using an Eppendorf Master Thermocycler (Hamburg, Germany). Amplification reactions were performed in a 25 μL reaction volume which contained 12.5 μL 2× Taq Plus Master Mix II (Vazyme, Nanjing, China), 1 μL of each forward and reverse primer (10 μM) (Tsingke, Qingdao, China), and 1 μL template genomic DNA in amplifier, and were adjusted with distilled deionized water to a total volume of 25 μL. PCR parameters were as follows: 94 °C for 5 min, followed by 35 cycles of denaturation at 94 °C for 30 s, annealing at a suitable temperature for 30 s, extension at 72 °C for 1 min and a final elongation step at 72 °C for 10 min. The annealing temperature for each gene was 52 °C for ITS and GS, 59 °C for CAL, 60 °C for GAPDH, 58 °C for ACT and CHS-1, 55 °C for TUB2. The PCR products were visualized on 1% agarose electrophoresis gel. Sequencing was conducted by the Tsingke Company Limited (Qingdao, China) bi-directionally. Consensus sequences were obtained using MEGA 7.0 (Kumar et al. 2016). All sequences generated in this study were deposited in GenBank (Table 2).

**Table 2.** Species and GenBank accession numbers of DNA sequences used in this study with new sequences in bold.

Species	Strain/Isolate	Host/Substrate	GenBank accession number						
			ITS	GAPDH	CHS-1	ACT	TUB2	CAL	GS
*Colletotrichumaenigma*	ICMP 18608*	*Perseaamericana*	JX010244	JX010044	JX009774	JX009443	JX010389	JX009683	JX010078
*C.aeschynomenes*	ICMP 17673*=ATCC 201874	*Aeschynomenevirginica*	JX010176	JX009930	JX009799	JX009483	JX010392	JX009721	JX010081
*C.alatae*	CBS 304.67*=ICMP 17919	*Dioscoreaalata*	JX010190	JX009990	JX009837	JX009471	JX010383	JX009738	JX010065
*C.alienum*	ICMP 12071*	*Malusdomestica*	JX010251	JX010028	JX009882	JX009572	JX010411	JX009654	JX010101
*C.aotearoa*	ICMP 18735	*Hedychiumgardnerianum*	JX010221	JX010023	JX009880	JX009500	JX010424	JX009620	JX010115
*C.arecicola*	hb8	*Arecacatechu*	MW561344	MW557464	-	-	MW557482	-	-
*C.artocarpicola*	MFLUCC18-1167*	*Artocarpusheterophyllus*	MN415991	MN435568	MN435569	MN435570	MN435567	-	-
*C.asianum*	ICMP 18580*=CBS 130418	*Coffeaarabica*	FJ972612	JX010053	JX009867	JX009584	JX010406	FJ917506	JX010096
*C.australianum*	BRIP 63695	*Capsicumannuum*	KU923677	MN442115	MW092000	MN442105	KU923693	-	KU923737
*C.boninense* (outgroup)	CBS 123755*	Crinumasiaticumvar.sinicum	JQ005153	JQ005240	JQ005327	JQ005501	JQ005588	-	-
*C.camelliae*	ICMP 10643	*Camellia×williamsi*	JX010224	JX009908	JX009891	JX009540	JX010436	JX009630	JX010119
*C.changpingense*	MFLUCC 15-0022*	*Fragaria×ananassa*	KP683152	KP852469	KP852449	KP683093	KP852490	-	-
*C.chiangmaiense*	MFLUCC 18-0945	*Magnoliagarrettii*	MW346499	MW548592	MW623653	MW655578	-	-	-
*C.chrysophilum*	CMM4268*	*Musa* sp.	KX094252	KX094183	KX094083	KX093982	KX094285	KX094063	KX094204
*C.ciggaro*	ICMP 19122	*Vaccinium* sp.	JX010228	JX009950	JX009902	JX009536	JX010433	JX009744	JX010134
*C.clidemiae*	ICMP 18658*	*Clidemiahirta*	JX010265	JX009989	JX009877	JX009537	JX010438	JX009645	JX010129
*C.cobbittiense*	BRIP66219	*Cordyline stricta × Cordyline australis*	MH087016	MH094133	MH094135	MH094134	MH094137	-	-
*C.conoides*	CAUG17*	*Capsicumannuum*	KP890168	KP890162	KP890156	KP890144	KP890174	-	-
*C.cordylinicola*	MFLUCC090551*=ICMP 18579	*Cordylinefruticosa*	JX010226	JX009975	JX009864	HM470235	JX010440	HM470238	JX010122
*C.dracaenigenum*	MFLUCC 19-0430*	*Dracaenafragrans*	MN921250	MT215577	MT215575	MT313686	-	-	-
*C.endophytica*	CAUG28	*Capsicumannuum*	KP145441	KP145413	KP145385	KP145329	KP145469	-	-
*C.fici-septicae*	MFLU 19-27708*	*Ficusseptica*	MW114367	MW183774	MW177701	MW151585	-	-	-
*C.fructicola*	MFLU 090228*	*Coffeaarabica*	FJ972603	FJ972578	-	FJ907426	FJ907441	FJ917508	FJ972593
*C.fructivorum*	CBS 133125*	*Vacciniummacrocarpon*	JX145145	-	-	-	JX145196	-	-
*C.gloeosporioides*	IMI356878*=ICMP 17821	*Citrussinensis*	JX010152	JX010056	JX009818	JX009531	JX010445	JX009731	JX010085
	ICMP 19121	*Citruslimon*	JX010148	JX010054	JX009903	JX009558	-	JX009745	-
	**SAUCC200952**	***Juglansregia***	**MW786743**	**MW876474**	**MW883689**	**MW883698**	**MW888973**	**MW922541**	**MW888964**
	**SAUCC200954**	***Juglansregia***	**MW786744**	**MW876475**	**MW883690**	**MW883699**	**MW888974**	**MW922542**	**MW888965**
	**SAUCC201001**	***Juglansregia***	**MW786745**	**MW876477**	**MW883692**	**MW883701**	**MW888976**	**MW922544**	**MW888967**
*C.grevilleae*	CBS 132879*	*Grevillea* sp.	KC297078	KC297010	KC296987	KC296941	KC297102	KC296963	-
*C.grossum*	CAUG7*	*Capsicum* sp.	KP890165	KP890159	KP890153	KP890141	KP890171	KP890147	-
*C.hebeiense*	MFLUCC130-726*	*Vitisvinifera*	KF156863	KF377495	KF289008	KF377532	KF288975	-	-
*C.hedericola*	MFLU 15-0689	*Hederahelix*	MN631384	-	MN635794	MN635795	-	-	-
*C.helleniense*	CBS 142418*	*Poncirustrifoliata*	KY856446	KY856270	KY856186	KY856019	KY856528	-	-
*C.henanense*	LF238*	*Camelliasinensis*	KJ955109	KJ954810	-	KM023257	KJ955257	KJ954662	KJ954960
*C.horii*	ICMP 10492	*Diospyroskaki*	GQ329690	GQ329681	JX009752	JX009438	JX010450	JX009604	JX010137
*C.hystricis*	CPC 28153*	*Citrushystrix*	KY856450	KY856274	KY856190	KY856023	KY856532	-	-
*C.jiangxiense*	LF687*	*Camelliasinensis*	KJ955201	KJ954902	-	KJ954471	KJ955348	KJ954752	KJ955051
*C.kahawae*	IMI 319418*=ICMP 17816	*Coffeaarabica*	JX010231	JX010012	JX009813	JX009452	JX010444	-	JX010130
*C.ledongense*	CGMCC3.18888*	*Quercuspalustris*	MG242008	MG242016	MG242018	MG242014	MG242010	-	-
*C.makassarense*	CBS 143664a*=CPC 28612	*Capsicumannuum*	MH728812	MH728820	MH805850	MH781480	MH846563	-	-
***C.mengyinense***	SAUCC200702*	***Rosachinensis***	**MW786742**	**MW846240**	**MW883686**	**MW883695**	**MW888970**	**MW922538**	**MW888961**
	**SAUCC200912**	***Juglansregia***	**MW786689**	**MW876472**	**MW883687**	**MW883696**	**MW888971**	**MW922539**	**MW888962**
	**SAUCC200913**	***Juglansregia***	**MW786690**	**MW876473**	**MW883688**	**MW883697**	**MW888972**	**MW922540**	**MW888963**
	**SAUCC200983**	***Juglansregia***	**MW786642**	**MW876476**	**MW883691**	**MW883700**	**MW888975**	**MW922543**	**MW888966**
*C.musae*	CBS 116870*=ICMP 19119	*Musa* sp.	JX010146	JX010050	JX009896	JX009433	HQ596280	JX009742	JX010103
*C.nupharicola*	CBS 470.96*=ICMP 18187	Nupharluteasubsp.polysepala	JX010187	JX009972	JX009835	JX009437	JX010398	JX009663	JX010088
*C.pandanicola*	MFLU 18-0003*	*Pandanus* sp.	MG646967	MG646934	MG646931	MG646938	MG646926	-	-
	**SAUCC200204**	***Juglansregia***	**MW786641**	**MW846239**	**MW883685**	**MW883694**	**MW888969**	**MW922537**	**MW888960**
	**SAUCC201152**	***Juglansregia***	**MW786746**	**MW876478**	**MW883693**	**MW883702**	**MW888977**	**MW922545**	**MW888968**
*C.perseae*	GA100*	*Perseaamericana*	KX620308	KX620242	-	KX620145	KX620341	KX620206	KX620275
*C.proteae*	CBS 132882*	*Protea* sp.	KC297079	KC297009	KC296986	KC296940	KC297101	KC296960	-
*C.pseudotheobromicola*	MFLUCC 18-1602	*Prunusavium*	MH817395	MH853675	MH853678	MH853681	MH853684	-	-
*C.psidii*	ICMP 19120	*Psidium* sp.	JX010219	JX009967	JX009901	JX009515	JX010443	JX009743	JX010133
*C.queenslandicum*	ICMP 1778*	*Caricapapaya*	JX010276	JX009934	JX009899	JX009447	JX010414	JX009691	JX010104
*C.rhexiae*	CBS 133134*	*Rhexiavirginica*	JX145128	-	-	-	JX145179	-	-
*C.salsolae*	ICMP 19051*	*Salsolatragus*	JX010242	JX009916	JX009863	JX009562	JX010403	-	-
*C.siamense*	ICMP 18578*	*Coffeaarabica*	JX010171	JX009924	JX009865	FJ907423	JX010404	FJ917505	JX010094
	ICMP 19118	*Jasminumsambac*	HM131511	HM131497	JX009895	HM131507	JX010415	-	JX010105
*C.syzygicola*	MFLUCC10-0624*	*Syzygiumsamarangense*	KF242094	KF242156	-	KF157801	KF254880	KF254859	-
*C.tainanense*	CBS 143666*	*Capsicumannuum*	MH728818	MH728823	MH805845	MH781475	MH846558	-	-
*C.temperatum*	Coll883*	*Vacciniummacrocarpon*	JX145159	-	-	-	JX145211	-	-
*C.theobromicola*	ICMP 18649	*Theobromacacao*	JX010294	JX010006	JX009869	JX009444	JX010447	JX009591	JX010139
*C.ti*	ICMP 4832*	*Cordyline* sp.	JX010269	JX009952	JX009898	JX009520	JX010442	JX009649	JX010123
*C.tropicale*	CBS 124949*=ICMP 18653	*Theobromacacao*	JX010264	JX010007	JX009870	JX009489	JX010407	JX009719	JX010097
*C.viniferum*	GZAAS5.08601*	*Vitisvinifera*	JN412804	JN412798	-	JN412795	JN412813	-	-
*C.wuxiense*	CGMCC 3.17894*	*Camelliasinensis*	KU251591	KU252045	KU251939	KU251672	KU252200	-	KU252101
*C.xanthorrhoeae*	BRIP 45094*=ICMP 17903 = CBS 127831	*Xanthorrhoeapreissii*	JX010261	JX009927	JX009823	JX009478	JX010448	JX009653	JX010138
*C.yulongense*	CFCC 50818*	*Vacciniumdunalianum*	MH751507	MK108986	MH793605	MH777394	MK108987	MH793604	MK108988
*Colletotrichum* sp.	BRIP 58074a	*Citrusaustralasica*	MK469999	MK470017	MW091975	MK470089	MK470053	-	MK470035

Strains marked with “*” are ex-type or ex-epitype.

### ﻿Phylogenetic analyses

Novel sequences were generated from the nine strains in this study, and all reference available sequences of *Colletotrichum* species were downloaded from GenBank. Multiple sequence alignments for ITS, GAPDH, CHS-1, ACT, TUB2, CAL and GS were constructed and carried out using the MAFFT v.7.11 online programme (http://mafft.cbrc.jp/alignment/server/, Katoh et al. 2019) with the default settings, and manually corrected where necessary. To establish the identity of the isolates at species level, phylogenetic analyses were conducted individually for each locus and then as combined analyses of seven loci (ITS, GAPDH, CHS-1, ACT, TUB2, CAL and GS). Phylogenetic analyses were based on maximum likelihood (ML) and Bayesian.

Inference (BI) for the multi-locus analyses. For BI, the best evolutionary model for each partition was determined using MrModeltest v. 2.3 (Nylander 2004) and incorporated into the analyses. ML and BI were run on the CIPRES Science Gateway portal (https://www.phylo.org/) using RaxML-HPC2 on XSEDE (8.2.12) (Miller et al. 2012; Stamatakis 2014) and MrBayes on XSEDE (3.2.7a), respectively (Huelsenbeck and Ronquist 2001; Ronquist and Huelsenbeck 2003; Ronquist et al. 2012). For ML analyses the default parameters were used and BI was carried out using the rapid bootstrapping algorithm with the automatic halt option. Bayesian analyses included seven parallel runs of 5,000,000 generations, with the stop rule option and a sampling frequency of 1000 generations. The burn-in fraction was set to 0.25 and posterior probabilities (PP) were determined from the remaining trees. The resulting trees were plotted using FigTree v. 1.4.4 (http://tree.bio.ed.ac.uk/software/figtree) and edited with Adobe Illustrator CS6.0. New sequences generated in this study were deposited at GenBank (https://www.ncbi.nlm.nih.gov; Table 2).

## ﻿Results

### ﻿Phylogenetic analyses

Nine strains of *Colletotrichum* isolated from leaves of *Rosachinensis* and fruit of *Juglansregia* in Mengyin County, Shandong Province, China, were grown in culture. Among the nine *Colletotrichum* isolates were identified a new species and two known species based on an analysis of combined ITS, GAPDH, CHS-1, ACT, TUB2, CAL and GS gene sequences composed of 69 isolates of *C.gloeosporioides* species complex and *C.boninense* (CBS 123755) as the outgroup taxon.

A total of 3953 characters including gaps were obtained in the phylogenetic analysis, viz. ITS: 1–619, GAPDH: 620–929, CHS-1: 930–1229, ACT: 1230–1542, TUB2: 1543–2288, CAL: 2289–3028, GS: 3029–3953. Of these characters, 2667 were constant, 674 were variable and parsimony-uninformative, and 612 were parsimony-informative.

**Figure 1.**
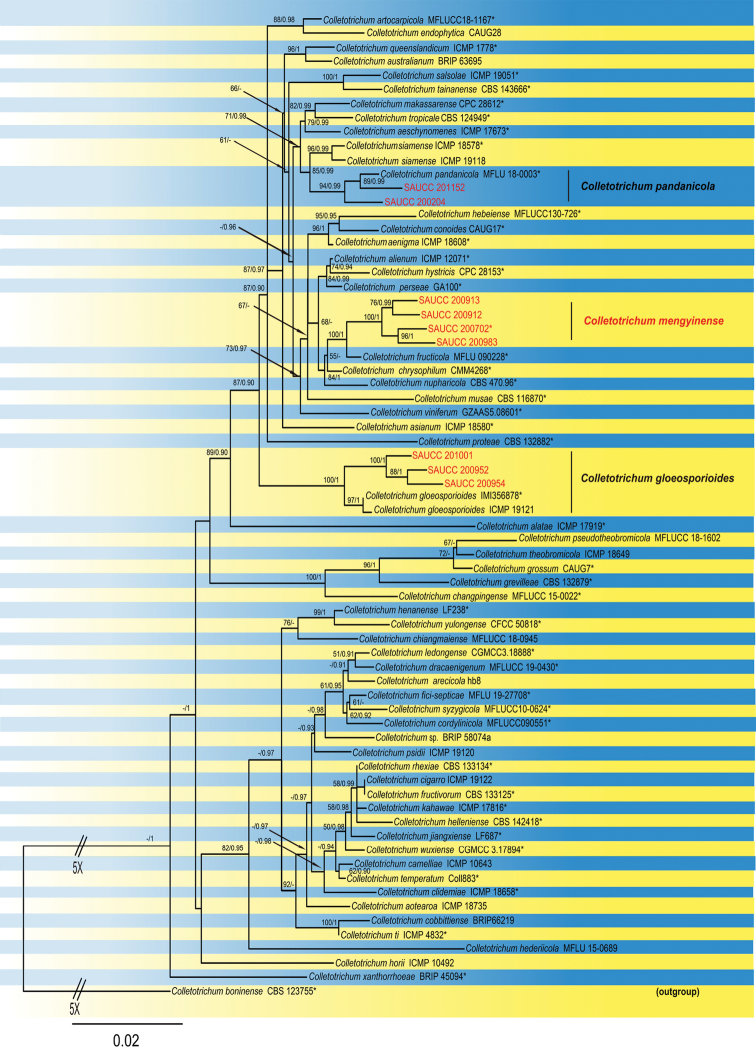
Phylogram of *Colletotrichumgloeosporioides* complex based on combined ITS, GAPDH, CHS-1, ACT, TUB2, CAL and GS genes. The ML and BI bootstrap support values above 50% and 0.90 BYPP are shown at the first and second position, respectively. Strains marked with “*” are ex-type or ex-epitype. Strains from this study are shown in red. Two branches were shortened to fit the page size-these are indicated by the symbol (//) with an indication number showing how many times they are shortened.

The Bayesian analysis lasted 4,685,000 generations, resulting in 4686 total trees, of which 3515 trees were used to calculate the posterior probabilities. The BI posterior probabilities were plotted on the ML tree. For the BI and ML analyses, HKY+G for GAPDH and ACT, SYM+I+G for ITS, K80+I+G for CHS-1, GTR+G for GS and CAL, HKY+I for TUB2 were selected and incorporated into the analyses. The ML tree topology confirmed the tree topologies obtained from the BI analyses, and therefore, the ML tree is presented (Fig. 1).

ML bootstrap support values (≥ 50%) and Bayesian posterior probability (≥ 0.90) are shown as first and second position above nodes, respectively. The 70 strains were assigned to 60 species clades based on the seven gene loci phylogeny (Fig. 1). The nine strains studied here represented a novel species and two known species. The new species of *C.mengyinense* showed a close relationship to *C.fructicola* (MFLU 090228) with full support (ML-BS: 100% and BYPP: 1). The strains SAUCC200952, SAUCC200954 and SAUCC201001 belong to *C.gloeosporioides* (IMI356878) with full support (ML-BS: 100% and BYPP: 1) by the multi-locus phylogeny. The strains SAUCC200204 and SAUCC201152 belong to *C.pandanicola* (MFLU 18-0003) with good support (ML-BS: 94% and BYPP: 0.99) by the multi-locus phylogeny.

### ﻿Taxonomy

#### ﻿ Colletotrichum gloeosporioides

Taxon classificationFungiGlomerellalesGlomerellaceae

(Penz.) Penz. & Sacc., Atti Reale Ist. Veneto Sci. Lett. Arti., ser. 6, 2: 670. 1884

DA5440E4-45FD-561B-B6CB-A7B91C558964

Figure 2

Vermicudaria gloeosporioides Penz., Michelia 2: 450, 1882. Basionym.

##### Description.

Lesion fruit, round or irregular, dark brown slightly sunken center, brown at margin. Asexual morph developed on PDA. A mass of orange conidia grows in the white mycelium of PDA after 14 days in light at 25 °C. Conidia, hyaline, smooth-walled, subcylindrical, both ends round, 1–3-guttulate, contents granular. Conidia on PDA (10.6–16.5 × 4.3–5.3 µm, mean ± SD = 14.9 ± 1.5 × 4.9 ± 0.3 μm, L/W ratio = 3.0, n = 40). Sexual morph not observed. Conidiogenous cells subcylindrical, straight to curved, 4.7–12.7 × 3.1–4.0 µm, opening 1.5–2.0 μm diam. Conidiophores hyaline, smooth walled, septate, branched.

**Figure 2.**
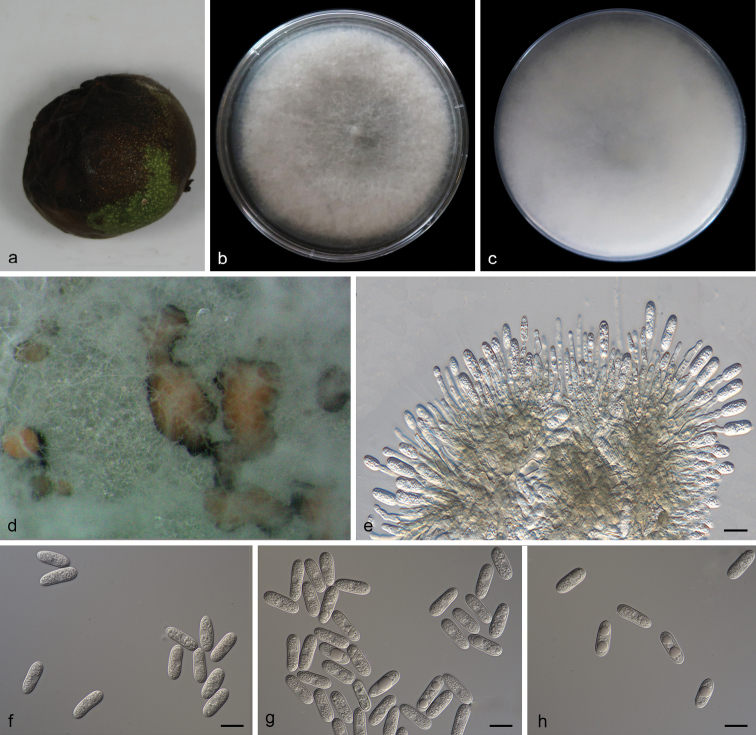
*Colletotrichumgloeosporioides* (SAUCC201001) **a** lesion fruit of host plant **b, c** surface (**b**) and reverse (**c**) sides of colony after incubation for 7 days on PDA **d** conidiomata **e** conidiophores, conidiogenous cells and conidia **f–h** conidia. Scale bars: 10 μm (**e–h**).

##### Culture characteristics.

Colonies on PDA flat with entire margin, aerial mycelium white, floccose cottony; surface and reverse grayish in the center and white margin. PDA attaining max 81 mm in diameter after 7 days, at 25 °C, growth rate 8.7–11.5 mm/day. Colonies on SNA sparse hyphae, slow growth.

##### Specimens examined.

China, Shandong Province: Mengyin County, Mengshan, on diseased fruit of *Juglansregia*, 25 July 2020, T.C. Mu, paratype HSAUP200952, ex-paratype living culture SAUCC200952. China, Shandong Province: Mengyin County, Mengshan, on diseased fruit of *Juglansregia*, 25 July 2020, T.C. Mu, paratype HSAUP200954, ex-paratype living culture SAUCC200954. China, Shandong Province: Mengyin County, Mengshan, on diseased fruit of *Juglansregia* 25 July 2020, T.C. Mu, paratype HSAUP201001, ex-paratype living culture SAUCC201001.

##### Notes.

*Colletotrichumgloeosporioides* was originally described as *Vermiculariagloeosporioides* on fruit of *Citrussinensis* in Italy and this species placed in *Colletotrichum* by Corda (Weir et al. 2012; Cannon et al. 2008). In the present study, three strains (SAUCC200952, SAUCC200954 and SAUCC201001) are clustered to *C.gloeosporioides* clade in the combined phylogenetic tree (Fig. 1). Morphologically, our strains were similar to *C.gloeosporioides* by conidia (10.6–16.5 × 4.3–5.3 vs. 12.0–17.0 (–23.5) × 4.5–6.0 μm, mean：14.9 × 4.9 vs. 14.4 × 5.6 μm). We therefore consider the isolated strain as *C.gloeosporioides*.

#### ﻿ Colletotrichum mengyinense

Taxon classificationFungiGlomerellalesGlomerellaceae

T.C. Mu, J.W. Xia, X.G. Zhang & Z. Li sp. nov.

8B12D86A-EE27-5999-8CF7-0A098B4F8771

841265

Figure 3

##### Etymology.

Named after Mengyin County where the fungus was collected.

##### Diagnosis.

*Colletotrichummengyinense* can be distinguished from the phylogenetically most closely related species *C.fructicola* (MFLU 090228) by its large conidia (12.5–15.7 × 4.8–6.1 vs. 9.7–14.0 × 3.0–4.3 μm), and five loci (2/509 in the ITS region, 1/139 GAPDH, 9/237 ACT, 8/410 TUB2 and 20/727 GS).

##### Type.

China, Shandong Province: Mengyin County, on diseased leaves of *Rosachinensis*, 25 July 2020, T.C. Mu, holotype HSAUP200702, ex-type living culture SAUCC200702.

##### Description.

Leaf spots discoid to irregular, brown or tanned. Asexual morph developed on SNA. A yellowish or orange mass appearing just as accumulations of conidia on the surface of the medium of SNA after 14 days in light at 25 °C. Conidia one-celled, hyaline, smooth-walled, subcylindrical, both ends round, contents granular. Conidia on SNA (12.5–15.7 × 4.8–6.1 µm, mean ± SD = 14.3 ± 1.1 × 5.3 ± 0.4 μm, L/W ratio = 2.7, n = 40). Sexual morph not observed. Conidiogenous cells subcylindrical, hyaline, 5.3–15.5 × 2.9–4.9 μm, opening 1.7–2.5 μm diam. Conidiophores hyaline, smooth walled, septate, branched.

**Figure 3.**
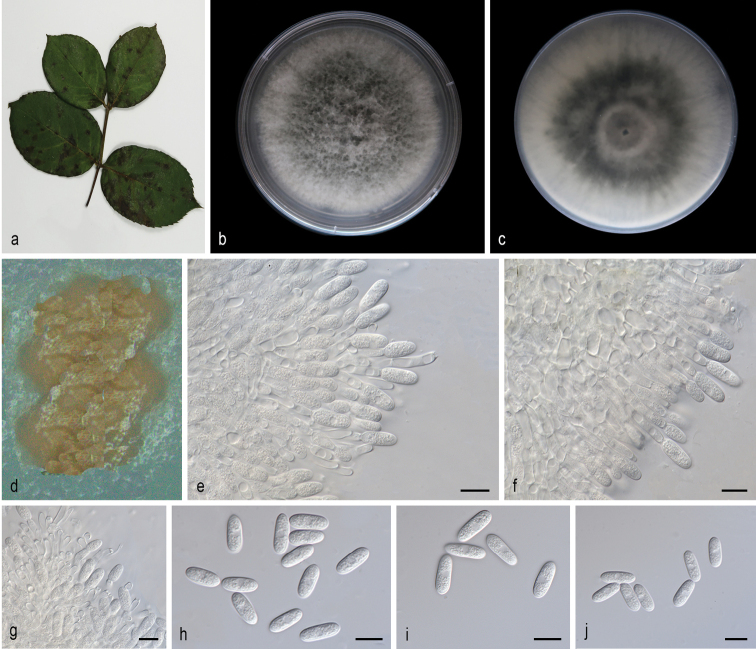
*Colletotrichummengyinense* (SAUCC200702) **a** branch with leaves of host plant **b, c** surface (**b**) and reverse (**c**) sides of colony after incubation for 7 days on PDA **d** conidiomata **e-g** conidiophores, conidiogenous cells and conidia **h–j** conidia. Scale bars: 10 μm (**e–j**).

##### Culture characteristics.

Colonies on PDA flat with entire margin, aerial mycelium white or gray, floccose cottony; surface and reverse gray in the center and grayish margin. PDA attaining 69.3–75.6 mm in diameter after 7 days, at 25 °C, growth rate 9.9–10.8 mm/day. Colonies on SNA sparse hyphae, slow growth.

##### Additional specimen examined.

China, Shandong Province: Mengyin County, on diseased fruit of *Juglansregia*, 25 July 2020, T.C. Mu, paratype HSAUP200912, ex-paratype living culture SAUCC200912. China, Shandong Province: Mengyin County, on diseased fruit of *Juglansregia*, 25 July 2020, T.C. Mu, paratype HSAUP200913, ex-paratype living culture SAUCC200913. China, Shandong Province: Mengyin County, on diseased fruit of *Juglansregia*, 25 July 2020, T.C. Mu, paratype HSAUP200983, ex-paratype living culture SAUCC200983.

##### Notes.

Phylogenetic analysis of a combined seven gene showed that *Colletotrichummengyinense* formed an independent clade (Fig. 1) and is phylogenetically distinct from *C.fructicola* (Prihastuti et al. 2009). This species can be distinguished from *C.fructicola* by 40 different nucleotides (2/509 in the ITS region, 1/139 in the GAPDH region, 9/237 ACT, 8/410 TUB2 and 20/727 GS). What’s more, *C.mengyinense* differs from *C.fructicola* in having large conidia (12.5–15.7 × 4.8–6.1 vs. 9.7–14.0 × 3.0–4.3 μm, mean: 14.3 × 5.3 vs. 11.53× 3.55 μm). Therefore, we establish this fungus as a novel species.

#### ﻿ Colletotrichum pandanicola

Taxon classificationFungiGlomerellalesGlomerellaceae

Tibpromma & K.D. Hyde, MycoKeys 33:47. (2018)

DFAE02E5-59C2-5685-BCD2-DB51E36B7D57

Figure 4

##### Description.

Lesion fruit, round or irregular, dark brown slightly sunken center, brown at margin. Asexual morph developed on SNA. A mass of yellowish or orange creamy conidial droplets at the inoculum point on SNA after 14 days in light at 25 °C. Born in conidiomata, conidia first take an ovoid shape, then become subcylindrical with rounded ends, contents granular. Conidia on SNA (14.2–17.9 × 4.6–6.0 µm, mean ± SD = 16.1 ± 0.9 × 5.4 ± 0.3 μm, L/W ratio = 2.9, n = 40). Sexual morph not observed. Conidiogenous cells subcylindrical, hyaline, 5.5–23.9 × 2.6–6.3 μm, opening 1.1–1.5 μm diam. Conidiophores branched, hyaline, smooth walled, septate, some septa disappeared at the end, contents granular.

**Figure 4.**
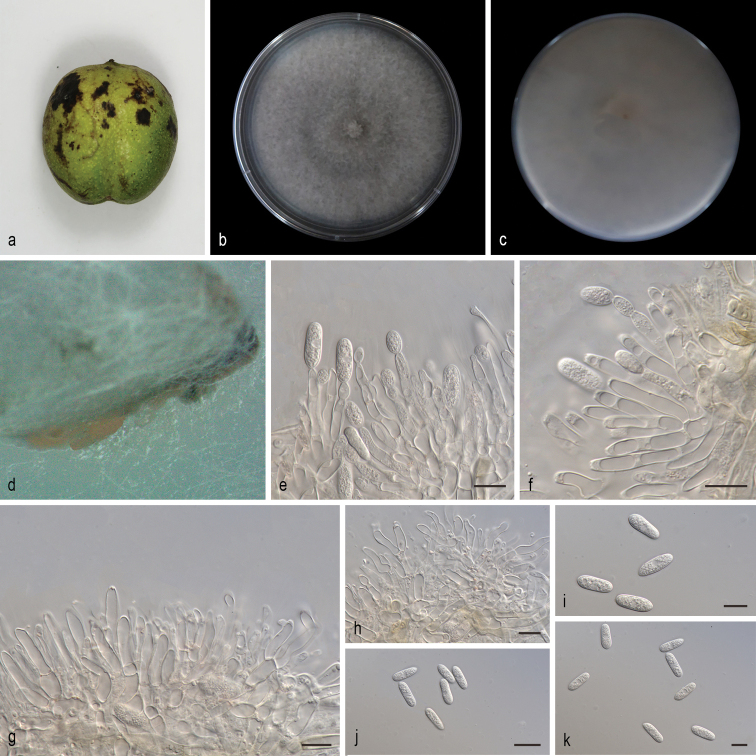
*Colletotrichumpandanicola* (SAUCC201152) **a** lesion fruit of host plant **b, c** surface (**b**) and reverse (**c**) sides of colony after incubation for 7 days on PDA **d** conidiomata **e, f** conidiophores, conidiogenous cells and conidia **g, h** conidiophores, conidiogenous cells **i–k** conidia. Scale bars: 10 μm (**e–k**).

##### Culture characteristics.

Colonies on PDA flat with entire margin, aerial mycelium white, floccose cottony; light gray in the center and pale white margin, reverse white to pale brownish. PDA attaining 58.1–82.6 mm in diameter after 7 days, at 25 °C, growth rate 8.3–11.8 mm/day. Colonies on SNA sparse hyphae, slow growth.

##### Specimens examined.

China, Shandong Province: Mengyin County, Mengshan, on diseased fruit of *Juglansregia*. 25 July 2020, T.C. Mu, paratype HSAUP200204, ex-paratype living culture SAUCC200204. China, Shandong Province: Mengyin County, Mengshan, on diseased fruit of *Juglansregia*. 25 July 2020, T.C. Mu, paratype HSAUP201152, ex-paratype living culture SAUCC201152.

##### Notes.

*Colletotrichumpandanicola* was originally described from the healthy leaves of *Pandanus* sp. (MFLU 18-0003, Pandanaceae) in Thailand (Tibpromma et al. 2018). In the present study, two strains (SAUCC200204 and SAUCC201152) are clustered to the *C.pandanicola* clade in the combined phylogenetic tree (Fig. 1). Morphologically, our strains were similar to *C.pandanicola* by conidia (14.2–17.9 × 4.6–6.0 vs. 9.0–18.0 × 4.0–8.0 μm, mean：16.1 × 5.4 vs. 13.39 × 5.35 μm). We therefore consider the isolated strains as *C.pandanicola*.

## ﻿Discussion

In this study, the *Colletotrichum* specimens of diseased leaves and fruits were collected in Mengyin, Shandong Province, China. A temperate monsoon climate and an abundance of fruit trees provide the proper conditions for anthracnose propagation. As a result, 70 reference sequences (including an outgroup taxon: *C.boninense* CBS 123755) were selected based on BLAST searches of NCBI’s GenBank nucleotide database and were included in the phylogenetic analyses (Table 2).

Phylogenetic analyses based on seven combined loci (ITS, GAPDH, CHS-1, ACT, TUB2, CAL and GS), as well as morphological characters of the asexual morph obtained in culture, were contributed to knowledge of the diversity of *Colletotrichum* species in Shandong Province. Based on a large set of freshly collected specimens from Shandong province, China, nine strains of *Colletotrichum* species were isolated from two host genera (Table 2). A new species is proposed: *C.mengyinense*. In a previous report, *C.gloeosporioides* has been isolated from *Juglansregia* (Zhu et al. 2014). *Colletotrichumpandanicola* was described from *Pandanus* sp. (Pandanaceae) in Thailand (Tibpromma et al. 2018) and *C.pandanicola* is first reported from *Juglansregia* in China. In this study, we described and illustrated *C.gloeosporioides* and *C.pandanicola* again.

Previously, species identification of *Colletotrichum* was largely referred to the host-specificity and pure culture characteristics, leading to the chaos of names (Weir et al. 2012). On the other hand, based on a polyphasic approach and known morphology, more than one species of *Colletotrichum* can colonize a single host, while one species can be associated with different hosts (Damm et al. 2012). It revealed diversity of *Colletotrichum* species from different hosts. Our study supported this result. For example, *C.pandanicola* (SAUCC200204 and SAUCC201152) and *C.gloeosporioides* (SAUCC200952, SAUCC200954 and SAUCC201001) were collected from *Juglansregia*. In addition, isolates of *C.mengyinense* were obtained from two hosts (*Juglansregia* and *Rosachinensis*). The morphological descriptions and molecular data for species of *Colletotrichum* represent an important resource and basis for plant pathologists and fungus taxonomists.

## Supplementary Material

XML Treatment for Colletotrichum gloeosporioides

XML Treatment for Colletotrichum mengyinense

XML Treatment for Colletotrichum pandanicola
